# Diagnosis of* Xeroderma Pigmentosum* Groups A and C by Detection of Two Prevalent Mutations in West Algerian Population: A Rapid Genotyping Tool for the Frequent* XPC* Mutation c.1643_1644delTG

**DOI:** 10.1155/2016/2180946

**Published:** 2016-06-20

**Authors:** Salima Bensenouci, Lotfi Louhibi, Hubert De Verneuil, Khadidja Mahmoudi, Nadhira Saidi-Mehtar

**Affiliations:** ^1^Laboratoire de Génétique Moléculaire et Cellulaire, Département de Génétique Moléculaire Appliquée, Faculté des Sciences de la Nature et de la Vie, Université des Sciences et de la Technologie d'Oran-Mohamed Boudiaf (USTO-MB), BP 1505, El M'naouer, 31000 Oran, Algeria; ^2^INSERM U1035, Biothérapies des Maladies Génétiques et Cancers, Université de Bordeaux, 146 rue Léo Saignat, 33000 Bordeaux Cedex, France; ^3^Service de Biochimie, Pôle de Biologie et Pathologie, Hôpital Pellegrin, Place Amélie Raba-Léon, 33000 Bordeaux Cedex, France; ^4^Service d'Ophtalmologie, Hôpital Pédiatrique de Canastel, rue du 1er Novembre, Bir El Djir, 31130 Oran, Algeria

## Abstract

*Xeroderma pigmentosum* (XP) is a rare autosomal recessive disorder. Considering that XP patients have a defect of the nucleotide excision repair (NER) pathway which enables them to repair DNA damage caused by UV light, they have an increased risk of developing skin and eyes cancers. In the present study, we investigated the involvement of the prevalent* XPA* and* XPC* genes mutations—nonsense mutation (c.682C>T, p.Arg228X) and a two-base-pair (2 bp) deletion (c.1643_1644delTG or p.Val548Ala fsX25), respectively—in 19 index cases from 19 unrelated families in the West of Algeria. For the genetic diagnosis of* XPA* gene, we proceeded to PCR-RFLP. For the* XPC* gene, we validated a routine analysis which includes a specific amplification of a short region surrounding the 2 bp deletion using a fluorescent primer and fragment sizing (GeneScan size) on a sequencing gel. Among the 19 index cases, there were 17 homozygous patients for the 2 bp deletion in the* XPC* gene and 2 homozygous patients carrying the nonsense* XPA* mutation. Finally,* XPC* appears to be the major disease-causing gene concerning* xeroderma pigmentosum *in North Africa. The use of fragment sizing is the simplest method to analyze this 2 bp deletion for the DNA samples coming from countries where the mutation c.1643_1644delTG of* XPC* gene is prevalent.

## 1. Introduction

*Xeroderma pigmentosum* (XP, OMIM 278700–278780) is a rare inherited autosomal recessive disorder characterized by an inability to repair DNA damage caused by ultraviolet (UV) light [1, 2] which induces skin cancers [3, 4] and other skin manifestations including poikiloderma, skin atrophy, telangiectasia, actinic keratoses, angiomas, and keratoacanthomas. XP patients may also have ocular manifestations, like photophobia, conjunctivitis, keratitis, ectropion, and entropion [5]. Neurologic symptoms such as mental deterioration, sensorineural deafness, hyporeflexia, and ataxia are found in severe forms of the disease [1, 6]. XP affects both sexes equally [7] with an incidence of 1/1 000 000 births in the USA and Europe [8], 1/20 000–100 000 in Japan [9, 10], and 1/10 000–30 000 in North Africa [11–14].

XP is found in all races worldwide and caused by defect in seven complementation groups (XP-A to XP-G) involved in NER system [15]. Four complementation groups (XP-A, XP-B, XP-D, and XP-G) exhibit neurological manifestations [16]. XP-C, XP-E, and XP-F patients rarely develop neurological disorders [17, 18]. Two different mechanisms of DNA repair can be distinguished: the Global Genome Repair (GGR) recognizes and removes lesions throughout the entire genome [19] while the Transcription-Coupled Repair (TCR) is specific to DNA damage occurring at transcribed strands of active genes [20]. Twenty per cent of XP patients present a normal NER system (XP-V) but a defective DNA polymerase eta (*η*) gene (POLH) [21]. XP-C and XP-A are the most prevalent groups in North Africa and Southern Europe [22, 23].

*Xeroderma pigmentosum* complementation group C (MIM ID #278720) is caused by mutations in the* XPC* gene (MIMID^*∗*^613208) and the most frequent mutation is a 2 bp deletion (c.1643_1644delTG, p.Val548AlafsX25) [13]. This gene is located on chromosome 3 (3p25) and contains 16 exons encoding for* xeroderma pigmentosum* group C (XPC) protein (GenBank accession number AC090645). This protein recognizes the damaged bases at the beginning of GGR pathway and it binds to HR23B to form the stable XPC-HR23B complex [24], which leads to the recruitment of TFIIH involved in the subsequent unwinding of the DNA double helix in concert with the proteins XPG, XPA, and replication protein A (RPA) [25].

*Xeroderma pigmentosum *complementation group A (MIM ID #278700) is caused by mutations in the* XPA* gene (MIMID^*∗*^ 611153) and the most frequent mutation is a nonsense mutation (c.682C>T, p.Arg228X) [26]. This gene is located on chromosome nine (9q34.1) [27] and consists of 6 exons that encodes for a 273-amino-acid Zn^2+^-finger protein [28]. The XPA protein plays a crucial role in both GGR and TCR pathways. The complex XPA-RPA provides the verification of the lesion and the binding to the single stranded DNA, so that the NER factors stay positioned around the lesion [29].

In the present study, we aimed to screen and detect for the first time the most common mutations in* XPA* and* XPC* genes presented in unrelated XP patients from the West of Algeria with clinical features of XP. Knowledge of these mutations is important for genetic counseling. Here, we used a new useful tool for rapid genotyping of the prevalent mutation of* XPC* gene.

## 2. Materials and Methods

### 2.1. Subjects

In this study, we collected 58 DNA samples from 19 unrelated XP families originated from Western Algeria. Among them, there were 19 index cases, 31 parents, and 8 siblings suffering from XP. Recruitment of patients with clinical diagnoses of XP disorder was provided at the Ophthalmology Department, Children's Hospital of Canastel, Oran (Algeria).

### 2.2. Methods

Informed signed consent for genetic investigation was obtained from all patients or from their parents in case of minors. Families were interviewed using a structured questionnaire to collect information about family history, consanguinity, affected members, and associated diseases. The clinical data of XP patients are summarized in Table 1.

#### 2.2.1. DNA Extraction

DNA was isolated from peripheral blood leukocyte using STRATAGENE kit reagents (Agilent Technologies Division) according to the manufacturer's instructions at the Laboratory of Molecular and Cellular Genetics.

#### 2.2.2. *XPA* Genotyping

To detect the previously reported nonsense mutation in exon 6 of the* XPA* gene (p.Arg228X), we used the following primers for the PCR reaction: forward primer 5′-TAC ATG GCT GAA AGC TTG AT-3′ and reverse primer 5′-GGG TTT CAT TCA TCT ATG-3′. The fragments were amplified by PCR in a volume of 50 *μ*L containing 50 ng of DNA, 1x PCR buffer, 0.2 mM of each dNTP, 2.5 mM MgCl_2_, 10 pmol of each one of the primers, and 1 U of Taq polymerase. PCR was performed using a Primus thermal cycler and the program included 95°C for 10 min, 35 cycles of 95°C for 30 s, 52°C for 30 s, and 72°C for 30 s. The cycles were followed by a final step of 72°C for 5 min. The PCR products were then subjected to RFLP analysis. Digestion of the PCR products was carried out according to the manufacturer's instructions. 5 *μ*L of the PCR products was digested overnight with 5 U of HphI at 37°C. The digestion products were separated on 2% agarose gel. The restriction enzyme HphI (New England Biolabs, USA) was used to distinguish the R228X mutation in which the gain of an HphI restriction site occurs in the mutant allele. The wild-type (C) has two bands (320 bp and 31 bp fragments); however, the mutant allele (T) has three bands (245 bp, 75 bp, and 31 bp fragments).

#### 2.2.3. *XPC* Genotyping

Specific amplification of the short region surrounding the deletion of two bases in exon 9 of the* XPC* gene was provided by two primers: a fluorescent forward primer 5′-(6FAM)GCATAGCTGGTATAGACCAG-3′ and a reverse primer 5′-gtttcttTCGTACCTCTGTGTGACATC-3′, which generates a fragment of 194 bp (normal allele) or 192 bp (deleted allele). PCR was performed in Primus thermal cycler using a final volume of 50 *μ*L containing 100 ng of DNA, 1x PCR buffer, 0.2 mM dNTPs, 2.5 mM MgCl_2_, 0.2 *μ*M of each one of the primers, and 1.25 U of Taq polymerase. PCR was performed as follows: 95°C for 10 min, 25 cycles of 94°C for 30 s, 30 s at 60°C, 30 s at 72°C, and a final step at 72°C for 5 min. The PCR products were checked on a 2% agarose gel. A step of purification and dilution was ensured for the PCR product with the addition of 90% formamide and 350 Rox marker in the loading dye. The fluorescent PCR products were separated on an automatic sequencer (ABI 3500, Applied Biosciences). Allele calling by fragment size analysis was performed with the aid of GeneScan software (Applied Biosciences). We also amplified the same exon 9 of* XPC* with different primers and sequencing products were analyzed by SeqScape software (Applied Biosciences).

## 3. Results

### 3.1. Clinical Findings

All investigated patients presented photophobia, skin photosensitivity, poikiloderma, and xeroderma with a mean age of 11 years. We registered consanguinity in 94.73% (18/19) of families. Among them, 88.89% were of the first degree and 11.11% of the second degree. XP symptoms had begun at a mean age of 16.5 months (range 2–36 months). Skin cancer was described in 73.68% of patients and ocular cancer was reported in 63.15%. Sex ratio (M/F) was 1.1, with different clinical presentations. Neurological symptoms were observed in 2 out of 19 patients. Besides data indicated in Table 1, we had also registered familial medical history, height, weight, and different common parameters.

### 3.2. Genotyping of XP-A Patients by PCR-RFLP and DNA Visualization by Gel Electrophoresis

Two XP patients (XP03 and XP05; see Table 1) who presented a clinical diagnosis of XP-A (association of different symptoms including neurological ones) were genotyped for the prevalent mutation* XPA* (p.Arg228X) in Pellegrin Hospital (Bordeaux). To detect the nonsense mutation in exon 6 of the* XPA* gene, we examined the HphI RFLP in the amplified exon 6 DNA fragments (351 bp). The C-to-T transition in exon 6 leads to a conversion of an arginine residue (CGA codon) to a stop codon (TGA) at amino acid 228. DNA from normal subjects who have a normal* XPA* allele gives a profile of two bands (320 bp and 31 bp fragments). In contrast, DNA from a XP patient with the homozygous mutation in exon 6 gives a profile of three bands (245 bp, 75 bp, and 31 bp fragments) caused by the creation of a new cleavage site for HphI in the 320 bp fragment by the nonsense mutation. After loading on 2% agarose gel, we found that both patients checked for the R228X mutation presented a profile homozygous for the mutant allele (T/T). Their parents were heterozygous carriers (C/T) with a profile of four bands (320 bp, 245 bp, 75 bp, and 31 bp fragments), confirming that these two patients are homozygous for the nonsense mutation at codon 228 and belong to the XP-A subclass.

### 3.3. Genotyping of XP-C Patients by Fragment Size and Sequence Analyses

**Figure 1:**
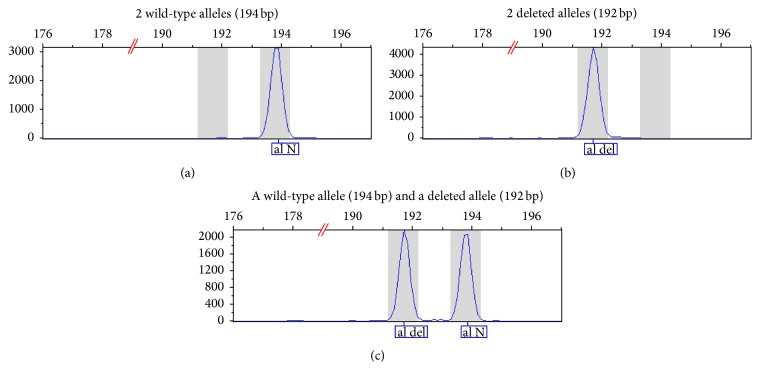
Characterization of the 2 bp deletion in exon 9 of the* XPC* gene. Fragment sizing analysis from electropherograms (GeneScan software) showed three possible profiles for the c.1643_1644delTG mutation: wild-type (a); homozygous deleted (b); heterozygous (c) profile.

**Figure 2:**
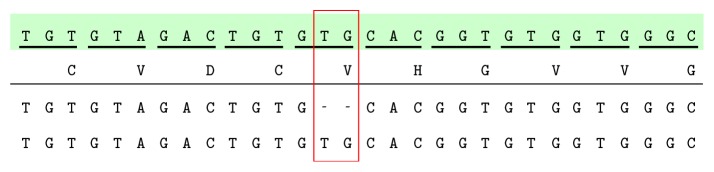
The corresponding sequence in the ninth exon of the* XPC* gene. The deduced amino acid sequence (NP_004619.3) in single letter code is shown below the reference sequence (NM_004628.4, highlighted in green). The 2 bp deletion is determined by a red rectangle.

**Figure 3:**
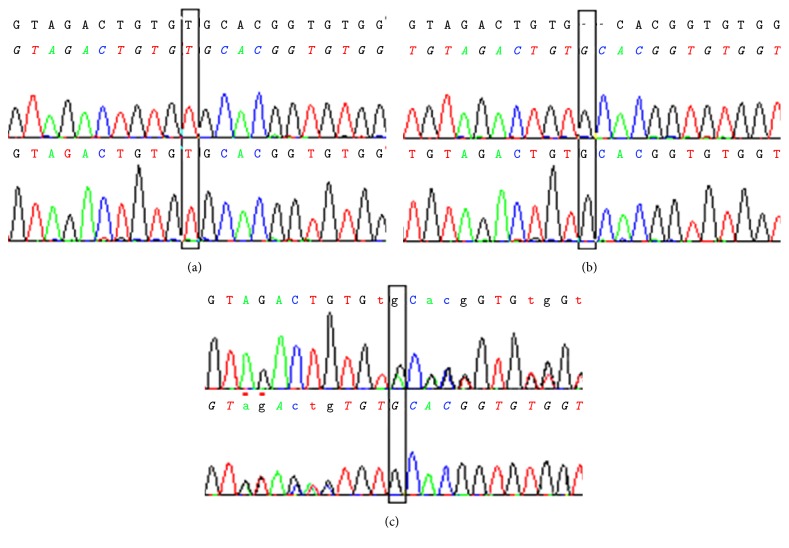
Sequence analysis of genomic DNA confirming the 2 bp deletion in the* XPC* gene. The alignment of sequences was obtained with the SeqScape software. The black rectangle indicates the position of the 2 bp deletion. Electropherograms show the three profiles: wild-type (a); homozygous deleted (b); heterozygous (c). For the c profile, because of the presence of both alleles, the two sequences (wild-type and deleted) were superimposed after the deletion.

Seventeen XP patients without neurological troubles (and their parents) were genotyped for the prevalent* XPC* mutation (c.1643_1644delTG, p.Val548AlafsX25) in Pellegrin Hospital. The GeneScan analysis performed for all samples tested showed three possible genotypes: wild-type (194 bp), heterozygous (192 and 194 bp), and homozygous (192 bp) for the deletion (Figure 1). All 17 index cases were homozygous for the 2 bp deletion. This deletion is responsible for a frame shift causing the occurrence of a premature stop codon 25 residues downstream; then we checked for the same mutation on their siblings and found that they were also homozygous for the 2 bp deletion. These results were confirmed by amplification of the fragment and sequencing: the presence of the deletion was identified by the SeqScape software (Applied Biosciences) (Figures 2 and 3).

## 4. Discussion

We report here a DNA analysis for 19 unrelated XP families. Our study focused on two frequent mutations, the frame shift mutation (c.1643_1644delTG, p.Val548AlafsX25), located in exon 9 of the* XPC* gene, and the nonsense mutation (c.682C>T) in exon 6 of the* XPA* gene, both already described.* XPA *mutations were present in 2 of 19 (10.5%) patients with the same nonsense mutation, c.682C>T (p.Arg228X), and* XPC* mutations were identified in 17 of 19 (89.5%) with the common* XPC* mutation, c.1643_1644delTG, present at the homozygous state in all XP-C patients. Obviously, there is a correlation between the phenotype and genotype, which highlights the importance of the precocious diagnosis in these patients and an early full protection against sun-exposure to allow them to have almost a normal life. The analysis of both parents (13 cases/19) or one parent (5 cases/19) showed that they were heterozygous carriers for the same mutation. In addition, the eight siblings suffering from XP were all homozygous for the frame shift mutation.

Molecular investigation of Soufir et al. [22] on 66 unrelated families from the Maghreb region showed that 85% of patients had mutations in the* XPC* gene; among them 87% shared the founder mutation (c.1643_1644delTG). 12% of XP patients had mutations in the* XPA* gene with a frequency of the mutation (c.682C>T) about 87.5%.

The XPA protein plays a central role in the first steps of NER and contains specific binding sites for other NER proteins such as DNA damage-binding protein 2 (DDB2) and RPA [30]. Hence, the severity of clinical manifestations decreases while the mutation moves from the N terminal to the C terminal of the protein, with the exception of the cases where splice site mutations permit the synthesis of small amount of normal protein [31]. Since the nonsense mutation is located on the sixth, N-terminal exon, we can speculate that most of the mRNA of the* XPA* gene would be produced, and it does not affect the major function of the* XPA* gene [32]. Of interest, the c.682C>T mutation occurs outside of the important domains such as the DNA-binding domain (exons 2–5, residues 98–219) and the zinc-finger motif (residues 105–129), but the XPA-TFIIH interaction region (residues 226–273  aa) essential for the excision reaction is lost, which explains the defective DNA repair system [26, 33, 34]. Therefore, the moderate phenotype of Tunisian XP-A patients may be explained by a residual DNA-binding activity of mutant XPA protein in comparison with the Japanese XP-A patients who had a splicing mutation at intron 3 at the homozygous state [26, 32]. A recent study of Tunisian XP-A patients showed the presence of a recurrent mutation R228X with a founder effect by haplotype analysis [26]. The R228X mutation was described in North Africa patients [22, 26, 32, 35].

The deficient XP-C human cells lead to a reduction of the cisplatin repair and increased mutagenesis [36]. This protein is a major factor in damage recognition to initiate global genome NER. The presence of the common 2 bp deletion leads to a premature termination codon and absence of normal XPC protein [37]. Indeed, it affects interaction capacities between XPC protein, HR23B, CETN2 (centrin-2 protein), and TFIIH molecules, which are necessary for DNA damage recognition [22]. The DNA repair ability was found to be only 20% of proficient normal cells with the presence of this previous mutation [38]. This same mutation was described by Ben Rekaya et al. [39] in 100% of 14 XP-C families from different regions of Tunisia and also in two other African patients with XP [40]. Interestingly, Mahindra et al. [41] also described the* XPC* mutation in two brother patients from North Sudan. Furthermore, the molecular analysis of 24 Moroccan patients showed that 17 were homozygous for the c.1643_1644delTG mutation [42]. Most XP-C African patients with delTG mutation in both alleles have similar clinical features consisting of photosensitivity, pigmentary lesions, and early onset of skin cancer without neurological involvement as our XP-C patients [22, 39, 41]. However, two patients were reported to be homozygous for the delTG but with neurological involvement [22].

Many studies showed that the mutation c.1643_1644delTG was spread in North Africa such as in Egypt, Italy, and Spain, which attracted the eyes of the researchers to the possibility of having the same ancestor. Soufir et al. proved the presence of a founder effect in the Mediterranean region using mathematical tools based on microsatellites haplotyping. They also showed that this common ancestor mutation was carried about 1250 years ago corresponding to 50 generations and approximately when Muslims from Arabia invaded Europe.

## 5. Conclusion

The high frequency of the founder mutation in XP patients from North Africa simplified the molecular diagnosis. In Algeria, the molecular investigation of XP confirmed that* XPC *and* XPA* genes were the most frequent with the mutations* XPC*-p.Val548AlafsX25 and* XPA*-R228X. Early diagnosis and full protection against sun-exposure are essential for preventing skin cancer and preserving vision in XP patients and can save lives.

It is important to do simple screening tests in risk families to detect heterozygous carriers for gene counseling, especially in communities with high marriage consanguinity. Moreover, prenatal diagnosis is greatly simplified when the molecular defect is easily accessible with the knowledge of the prevalent mutation in a given population, as observed in XP-C and XP-A patients.

Considering the efforts and costs required for the unscheduled DNA synthesis on cultured fibroblasts, we therefore recommend the use of DNA fragment sizing as the simplest and faster method for screening in XP diagnosis. In the population tested for this study, the high level of consanguinity, the large families with numerous siblings affected, and a high rate of premature death lead to the conclusion that the disease is clearly a major health concern. This simple screening test in risk families may greatly facilitate genetic counseling in exposed families as well as early management of affected patients. Furthermore, early diagnosis may improve prognosis of the disease, with extensive protection against the sun.

## Figures and Tables

**Table 1:** Clinical features of the nineteen patients with *xeroderma pigmentosum*.

Patient (code)	Region of origin in Algeria	Sex	Age	Age of onset (months)	Consanguinity	Clinical symptoms			
						Photophobia	Skin lesions	Tumors	Neurological abnormalities
XP01	Northwest	M	17	12	1stD	+	+	++	−
XP02	Northwest	F	12	7	1stD	+	+	+++	−
XP03	Northwest	F	7	2	1stD	+	+	−	+
XP04	Northwest	F	15	12	1stD	+	+	+	−
XP05	Northwest	M	8	3	1stD	+	+	+	+
XP06	Northwest	M	11	24	2ndD	+	+	++	−
XP07	Northwest	M	15	12	1stD	+	+	+++	−
XP08	Northwest	F	10	36	1stD	+	+	+	−
XP09	Northwest	M	14	8	1stD	+	+	+++	−
XP10	Northwest	F	13	36	1stD	+	+	+++	−
XP11	Northwest	M	23	12	1stD	+	+	+++	−
XP12	Middle West	M	4	24	1stD	+	+	−	−
XP13	Northwest	M	10	12	1stD	+	+	−	−
XP14	Northwest	F	4	12	1stD	+	+	+	−
XP15	Northwest	F	6	18	1stD	+	+	++	−
XP16	Northwest	M	15	36	1stD	+	+	+++	−
XP17	Middle West	M	25	12	AC	+	+	+++	−
XP18	Southwest	F	6	24	1stD	+	+	−	−
XP19	Middle West	F	6	11	2ndD	+	+	−	−

1stD: consanguinity first degree; 2ndD: consanguinity second degree; AC: absence of consanguinity.

−: absence of clinical symptoms; +: presence of clinical symptoms.
